# Outcomes of phase I clinical trials for patients with advanced pancreatic cancer: update of the MD Anderson Cancer Center experience

**DOI:** 10.18632/oncotarget.19897

**Published:** 2017-08-03

**Authors:** Jennifer B. Goldstein, Chad Tang, Kenneth R. Hess, David Hong, Vivek Subbiah, Filip Janku, Siqing Fu, Daniel D. Karp, Aung Naing, Apostolia Maria Tsimberidou, Jennifer Wheler, Ralph Zinner, Milind Javle, Gauri R. Varadhachary, Robert A. Wolff, David R. Fogelman, Funda Meric-Bernstam, Sarina A. Piha-Paul

**Affiliations:** ^1^ Division of Cancer Medicine, The University of Texas MD Anderson Cancer Center, Houston, TX, USA; ^2^ Department of Radiation Oncology, The University of Texas MD Anderson Cancer Center, Houston, TX, USA; ^3^ Department of Biostatistics, The University of Texas MD Anderson Cancer Center, Houston, TX, USA; ^4^ Department of Investigational Cancer Therapeutics, The University of Texas MD Anderson Cancer Center, Houston, TX, USA; ^5^ Department of Gastrointestinal Medical Oncology, The University of Texas MD Anderson Cancer Center, Houston, TX, USA

**Keywords:** pancreatic cancer, phase I trial, biomarker, chemotherapy, targeted therapy

## Abstract

**Background:**

In 2011, we reported the outcomes of pancreatic cancer (PC) patients enrolled in phase I trials at our institution from 2004 through 2009. At the time, gemcitabine and erlotinib were the only Food and Drug Administration-approved drugs for PC and median overall survival (OS) from consultation in the phase I clinic was 5 months. We sought to determine the impact of novel therapeutics on PC patients in phase I trials.

**Methods:**

We reviewed records of PC patients treated in phase I trials at our institution from January 2009 through December 2014. Survival was analyzed using the Kaplan-Meier method.

**Results:**

Ninety-five patients were identified. The median age was 61 years (range, 40-84), 59% were men, and 41% had stage IV disease. The median OS from consultation in the phase I clinic was 5.8 months (95% confidence interval [CI], 4.5-6.8), and the 1-year OS rate was 9% (95% CI, 4%-17%). Three patients had partial responses and 18 had stable disease ≥ 4 months.

**Conclusion:**

We observed no improvement in OS between PC patients enrolled in phase I trials in 2004-2009 and 2009-2015. To substantially improve OS in this challenging disease, improved patient selection and science-driven, innovative trial designs will be key.

## INTRODUCTION

Over the last 30 years, breakthroughs in chemotherapy, targeted therapy, and immunotherapy have led to progress in cancer treatment. Despite these advances, the mortality rate of pancreatic cancer has remained relatively stagnant. According to the United States National Cancer Institute's Surveillance, Epidemiology, and End Results database, between 1975 and 2006, the 5-year pancreatic cancer survival rate improved only minimally and remained at less than 5% [1]. The large majority of patients with pancreatic ductal adenocarcinoma (PDAC), 75-80%, present with advanced disease and are candidates for palliative systemic therapy. In the 25% of patients who present with resectable disease, surgery is the only potentially curative option, and among patients who undergo potentially curative surgery, the disease recurs within 3 years in 75% of patients [2]. Only 7 agents are currently approved by the US Food and Drug Administration for pancreatic cancer, and only 3 of these were approved within the last 5 years [3–8]. More recently, combination chemotherapy has been shown to moderately improve survival over single-agent gemcitabine in patients with pancreatic cancer. The PRODIGE study reported a median overall survival (OS) of 11.1 months with FOLFIRINOX (5-fluouracil, oxaliplatin, and irinotecan) versus 6.8 months with single-agent gemcitabine [9], and the MPACT study reported a median OS of 8.5 months with nab-paclitaxel plus gemcitabine versus 6.7 months with single-agent gemcitabine [7]. Erlotinib, although approved for treatment of PDAC, is not often used because it has demonstrated no clinically meaningful benefit [6]. In 2016, in the NAPOLI study, in which patients were randomly assigned to treatment with nanoliposomal irinotecan or 5-fluouracil, median OS was 6.1 months with irinotecan versus 4.2 months with 5-fluouracil [10]. This study established nanoliposomal irinotecan as another option for treatment of metastatic pancreatic cancer; however, treatment options remain very limited.

In 2011, we reported our experience with pancreatic cancer patients enrolled in phase I clinical trials from November 2004 to March 2009 [11]. At the time, the only standard-of-care agents available for treatment of metastatic pancreas cancer were gemcitabine and erlotinib, and promising clinical trial options were limited. In that previous study, we found that the median OS from first consultation in the phase I clinic was 5 months, and the median OS from the time of diagnosis was 22.1 months [11]. The long median OS in this population was attributed to good performance status, intact organ function, and selection bias. Forty-three of the 83 patients in that study (52%) were enrolled in a trial of oral curcumin, thought to act as a suppressor of nuclear factor κB and angiogenesis. Since our previous study, there have been multiple discoveries in the arena of targeted therapy and immunotherapy for cancer. The impact of these new agents on outcomes of patients with pancreatic cancer has not been systematically analyzed.

The aim of this study was to assess the impact of recently developed cancer therapeutics, including targeted therapies and immunotherapies, on outcomes of patients with pancreatic cancer in phase I clinical trials. We performed a retrospective review of patients who enrolled in phase I clinical trials from January 2009 through December 2014 at The University of Texas MD Anderson Cancer Center's Department of Investigational Cancer Therapeutics.

## RESULTS

### Patient characteristics

Patient characteristics are summarized in Table 1. Approximately 400 pancreatic cancer patients were seen per year at MD Anderson from January 2009 through December 2014. Of these, 95 patients with pancreatic adenocarcinoma (approximately 4% of patients seen) were treated in phase I clinical trials. These patients were treated on 52 different clinical trials. The median age of the patients was 61 years (range, 40-84 years). The study included 56 men and 39 women. Seventy patients were white (74%), 12 were black (13%), 6 were Hispanic (6%), and 5 were Asian (5%). At diagnosis of pancreatic cancer, 56 patients (59%) had stage I-III disease, and 39 patients (41%) had stage IV disease. The cancer was well to moderately differentiated in 34 patients (36%), and poorly differentiated in 28 patients (30%); 32 patients (34%) had an unknown tumor grade. At first consultation in the phase I clinic, 92 patients (97%) had metastatic disease and 24 patients (25%) had ascites. The median documented performance status score at the start of phase I treatment was 1 (range, 0-2). Five patients were either alive or lost to follow-up at the time of the analysis.

**Table 1:** Patient characteristics

Characteristic	No. of patients (%)(N = 95)
Age at start of best phase I trial, median (range), years	61 (range, 40-84)
Sex	
Male	56 (59)
Female	39 (41)
Race/ethnicity	
White	70 (74)
Black	12 (13)
Hispanic	6 (6)
Asian	5 (5)
Other	2 (2)
Stage at diagnosis	
I	7 (7)
II	32 (34)
III	17 (18)
IV	39 (41)
Tumor grade	
Well to moderately differentiated	34 (36)
Poorly differentiated	28 (29)
Other^*^ Unknown	1 (1)32 (34)
Location of tumor	
Head/neck	58 (61)
Body	17 (18)
Tail	20 (21)
Performance status at start of best phase I trial	
0	27 (28)
1	32 (34)
2	6 (6)
No. of prior treatments, median (range)	3 (1-8)
≥ 5 prior treatments	23 (24)
Ascites at presentation	24 (25)
Prior thrombotic event	
None	73 (77)
Pulmonary embolism	9 (10)
Deep vein thrombosis	7 (7)
Portal vein thrombosis	3 (3)
Stroke	1 (1)
Other	2 (2)

^*^One patient had a tumor with well-differentiated, moderately-differentiated, and poorly-differentiated components.

### Treatment

The median time from diagnosis to treatment on a phase I protocol was 14 months (range, 0.2-119). Prior to referral to our phase I clinic, patients had been treated with a mean of 3 regimens (range, 1-8) and 23 patients (24%) had been treated with at least 5 prior treatments. (Regimens included systemic chemotherapy or chemoradiation given in the neoadjuvant, adjuvant or metastatic settings, but did not include surgical resection or radiation alone.)

In contrast, in our previous study, published in 2011, patients had been treated with a mean of 2 regimens [11]. All 95 patients in our current study had been treated with chemotherapy and 35 patients (37%) had been treated with radiation therapy prior to referral to our phase I clinic. Thirty-five patients (37%) had received FOLFIRINOX, and 24 patients (25%) had received gemcitabine plus nab-paclitaxel. Thirty-four patients (36%) had undergone pancreatectomy or partial pancreatectomy, versus 39% in our 2011 study. All surgical operations were performed prior to referral to our phase I clinic.

The phase I clinical trials in which patients were enrolled are summarized in Table 2 and Supplementary Table 1. In these phase I trials, 30 patients were treated with cytotoxic chemotherapy alone, 51 patients were treated with targeted agents, 39 patients were treated with anti-VEGF therapy, and 5 patients were treated with immunotherapy.

**Table 2:** Summary of best phase I clinical trials

Treatment	Mechanism of action	No. of patients(%)(N = 95)	Mean time on study, months
Gemcitabine	Nucleoside analog	15 (16)	3.93
Nab-paclitaxel	Microtubule stabilization		
Bevacizumab	VEGF inhibition		
Bevacizumab	VEGF inhibition		
Trastuzumab	HER2/neu inhibition		
Lapatinib	HER2/neu and EGFR inhibition	13 (14)	3.23
Oxaliplatin by hepatic arterial infusion	DNA crosslinking	5 (5)	2.4
Capecitabine	Nucleoside analog		
Wnt inhibitor		5 (5)	1
Hydroxychloroquine with	Autophagy inhibition		
Vorinostat	Histone deacetylase inhibition	3 (3)	0.74
Sirolimus	mTOR inhibition	2 (2)	1.2
Aurora kinase/VEGF inhibitor		4 (4)	1.25
Bevacizumab/temsirolimus with	VEGF inhibition/calcineurin inhibition		
Paclitaxel	Microtubule stabilization	1 (1)	1.33
Carboplatin	DNA crosslinking	1 (1)	1.17
Sorafenib	CRAF, BRAF, VEGFR2, VEGFR3, PDGFR beta inhibition	2 (2)	0.95
Other^*^		44 (47)	3.13

BRAF, B-type Raf kinase; CRAF, C-type Raf kinase; EGFR, epidermal growth factor receptor; mTOR, mammalian target of rapamycin; nab, nanoparticle albumin-bound; PDGFR, platelet derived growth factor receptor; VEGF, vascular endothelial growth factor; VEGFR, vascular growth factor receptor.

^*^Other regimens are summarized in Supplementary Table 1.

### Response

**Figure 1:**
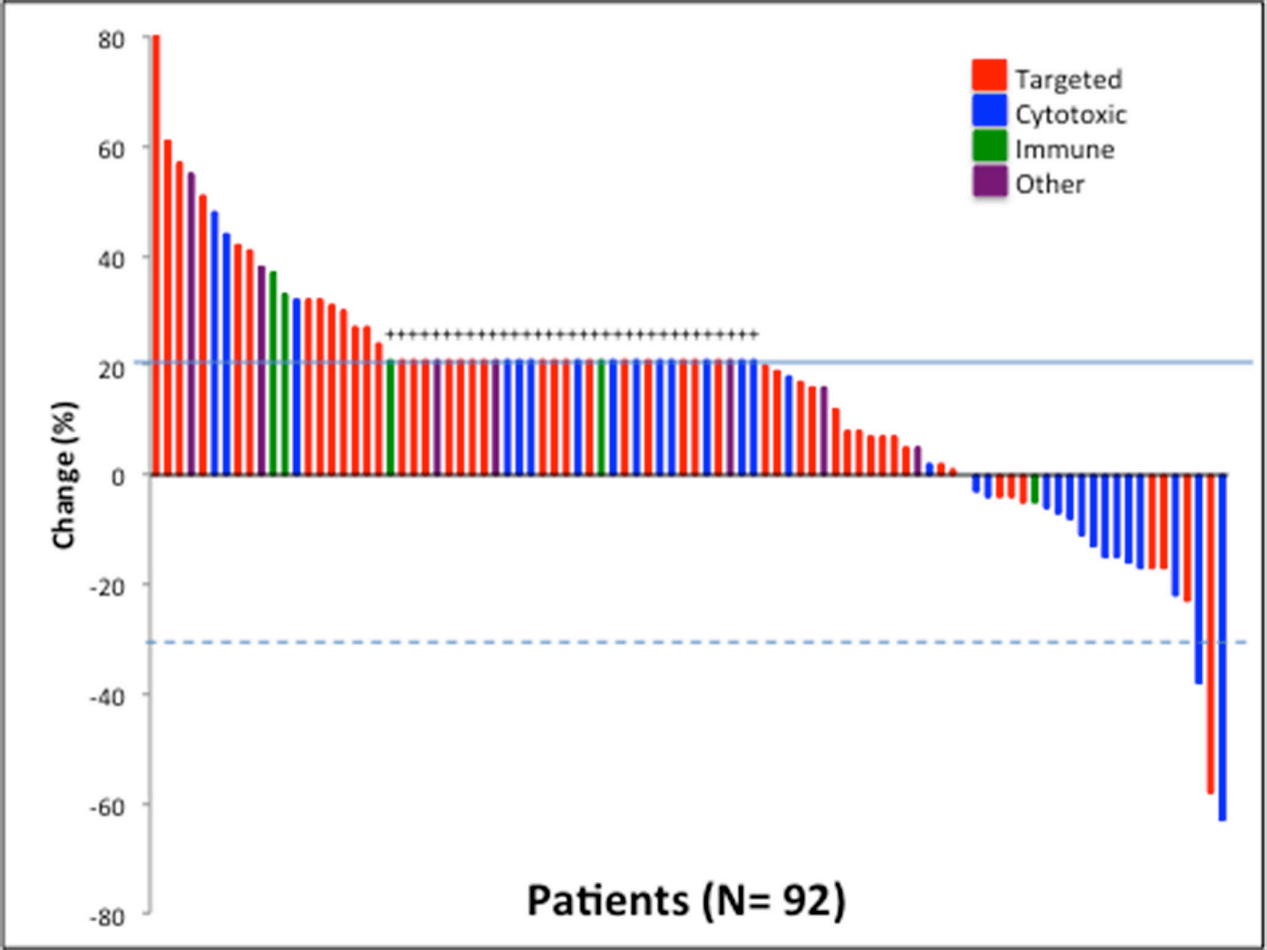
Waterfall plot depicting best RECIST response by patient Individual patients are represented by vertical bars on the X-axis. The best RECIST response (%) is depicted on the Y-axis. Ninety-two of the 95 patients had response measurable by RECIST. Thirty-three patients were assigned a value of +21% (progressive disease) because of clinical progression or new lesions (+). Solid line shows 20% progression by RECIST. Dotted line shows 30% response by RECIST.

Of the 95 patients assessed, 92 were evaluable for response with at least 1 post-baseline imaging study available. Three patients were not evaluable for response, one patienthad an allergic reaction to therapy and was taken off protocol for toxicity, one patienthad myocardial infarction deemed unrelated to the trial, and one patient had intracranial hemorrhage deemed unrelated to the trial. The 92 patients evaluable for response were treated on a total of 39 clinical trials for best phase I response analysis. Best phase I response was defined as the best response recorded from the start of the study treatment until the end of treatment of all of the phase I trials that the patient participated in. The median duration on regimen with best phase I response was 1.9 months (range, 0.2-21.3). The best responses were a partial response (≥ 30% reduction in tumor volume) in 3 patient (3%) and stable disease lasting at least 4 months in 18 patients (20%) (Figure 1). Eleven of the 21 patients (52%) with a partial response or stable disease lasting at least 4 months were treated with regimens that included bevacizumab on their best phase I clinical trial. Ten patients (11%) had a partial response or stable disease lasting at least 6 months. Our previous study of patients with pancreatic cancer treated in phase I trials demonstrated partial responses in 3% of patients and stable disease lasting at least 4 months in 13%. Of the 5 patients treated with immunotherapy, 4 had progressive disease, and 1 patient, treated with an anti-PD1 antibody, had stable disease lasting for a little over 4 months.

### Survival

**Figure 2:**
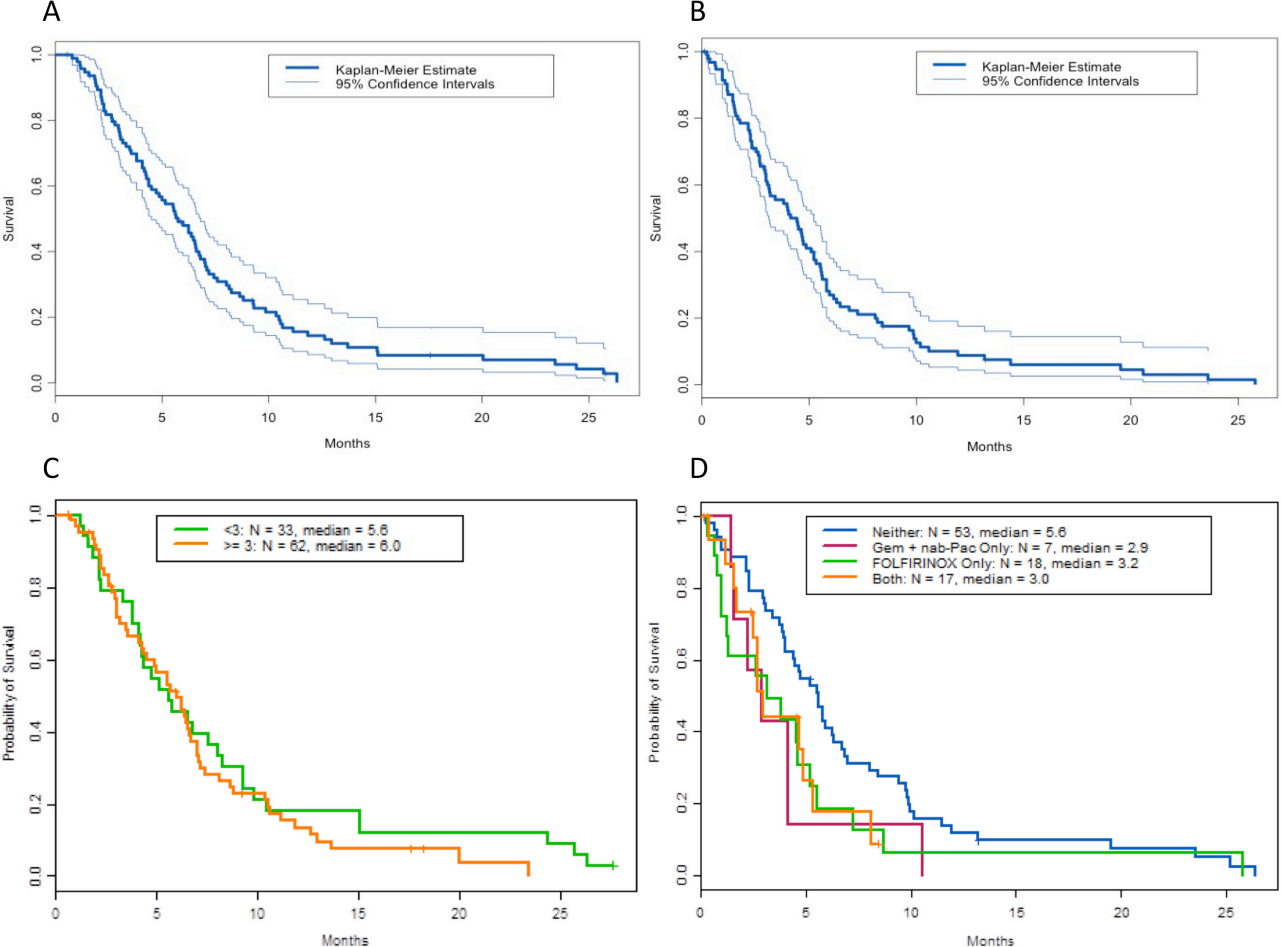
Overall survival (OS) of patients treated in phase I clinical trials Shown are Kaplan Meier curves of **(A)** OS from first consultation in the phase I clinic (N=95), **(B)** from initiation of best phase I treatment (N = 95), **(C)** OS from referral to our phase I clinic based on number of therapies prior to referral to the phase I clinic, and **(D)** OS from initiation of first phase I trial based on prior treatment with 5-fluorouracil, oxaliplatin, and irinotecan (FOLFIRINOX), gemcitabine (Gem) plus nab-paclitaxel (nab-Pac).

At the time of analysis, 87 patients (92%) had died, 5 had been lost to follow-up, and 1 was living. The median progression-free survival from consultation in the phase I clinic was 1.9 months (data not shown). Twenty-seven patients had a progression-free survival duration of at least 3 months. The median OS from first consultation in our to phase I clinic was 5.8 months (95% confidence interval [CI], 4.5-6.8 months, Figure 2A), compared to 5.0 months reported in the 2011 study [11]. The median OS from initiation of the first phase I treatment was 4.5 months (95% CI, 3.8-5.5), and the 1-year OS survival rate from the first phase I treatment was 9% (95% CI, 4%-17%; data not shown). The median OS from the initiation of the best phase I treatment was 4.1 months (95% CI, 3.1%-5.2%, Figure 2B), and the median OS from the date of diagnosis was 20.6 months (95% CI, 17-24.1), similar to the 22.1 months reported in our previous study.

The median OS from first consultation in our phase I clinic was 3.8 months in patients with and 5.2 months in patients without ascites at presentation (*P*= 0.08, hazard ration [HR], 1.5 [95% CI, 1.0-2.3]; data not shown). The median OS from first consultation in our phase I clinic was similar in patients treated with anti-VEGF therapy with current standard-of-care agents, patients treated with anti-VEGF therapy without standard-of-care agents, and patients not treated with anti-VEGF therapy (5.8 months, 6.0 months, and 5.0 months, respectively; *P* = 0.78; HR, 1.1 [95% CI, 0.7-1.9] for anti-VEGF therapy with standard-of-care vs. no anti-VEGF therapy and HR, 1.2 [95% CI, 0.7-2.1] for anti-VEGF therapy without standard-of-care vs. no anti-VEGF therapy; data not shown).

Sixty-two percent of patients (57 of 92) in the current study were treated with novel agents, compared to 92% of patients (76 of 83) in our previous study. The median OS from the initiation of the first phase I trial was 4.2 months in patients treated with novel therapies and 5.2 months in patients treated with a standard-of-care chemotherapeutic backbone agent (i.e. 5-fluorouracil or gemcitabine) (*P* = 0.76; HR, 1.1 [95% CI, 0.7-1.7]; data not shown).

The median OS from referral to the phase I clinic was 5.6 months for patients with fewer than 3 regimens prior to referral to our phase I clinic and 6.0 months for patients with three or more therapies prior to referral (*P* = 0.40; HR, 1.2 [95% CI 0.8-1.9]; Figure 2C).

Patients with prior exposure to FOLFIRINOX or gemcitabine plus nab-paclitaxel prior to phase I referral did not appear to have longer survival from date of diagnosis than patients not treated with these regimens (3.2 months, 2.9 months, 5.6 months respectively; *P* = 0.93; HR, 1.7 [95% CI 1.0-2.9] for FOLFIRINOX vs. neither and HR, 1.9 [95% CI 0.9-4.3] for gemcitabine plus nab-paclitaxel; Figure 2D).

### Multivariate analysis

A Cox proportional hazards model was used to assess the impact of multiple factors on OS from first phase I treatment. The results are shown in Table 3. Prior treatment with FOLFIRINOX (*P*= 0.046, HR 1.73, 95% CI 1.01-2.98) and liver metastases (*P*= 0.045, HR 1.72, 95% CI 1.01-2.91) were associated with a significantly worse OS. There was a trend towards association of prior radiation therapy (*P*= 0.077, HR 1.58, 95% CI 0.95-2.61) with worse OS. Location of the tumor at presentation (body vs. head/neck: *P* = 0.64, HR, 1.1 [95% CI, 0.6- 2.0]; tail vs. head/neck: *P* = 0.11, HR, 0.6 [95% CI, 0.4-1.1]) and prior history of thromboembolic event did not impact survival (*P* = 0.63, HR, 0.9 [95% CI 0.5-1.5]; data not shown).

**Table 3:** Results of multivariate Cox proportional hazards regression analysis performed to assess the impact of multiple factors on overall survival (OS) from first phase I treatment

Variable	Contrast	Hazard ratio (95% CI)	*P v*alue
ECOG performance status	> 0 vs. 0	1.47 (0.90, 2.41)	0.12
Liver metastases	Yes vs. No	1.72 (1.01, 2.91)	0.045
No. of metastatic sites	>2 vs. <= 2	1.33 (0.9, 2.25)	0.28
Prior radiation therapy	Yes vs. No	1.58 (0.95, 2.61)	0.077
Prior FOLFIRINOX	Yes vs. No	1.73 (1.01, 2.98)	0.046
Prior gemcitabine plus nab-paclitaxel treatment	Yes vs. No	1.08 (0.58, 2.01)	0.80

FOLFIRINOX, 5-fluorouracil, oxaliplatin, and irinotecan.

### Targeted therapy

**Figure 3:**
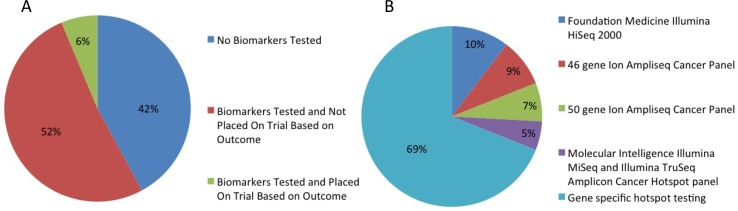
**(A)** Percent of patients undergoing biomarker testing, **(B)** type of platform used for biomarker testing.

**Figure 4:**
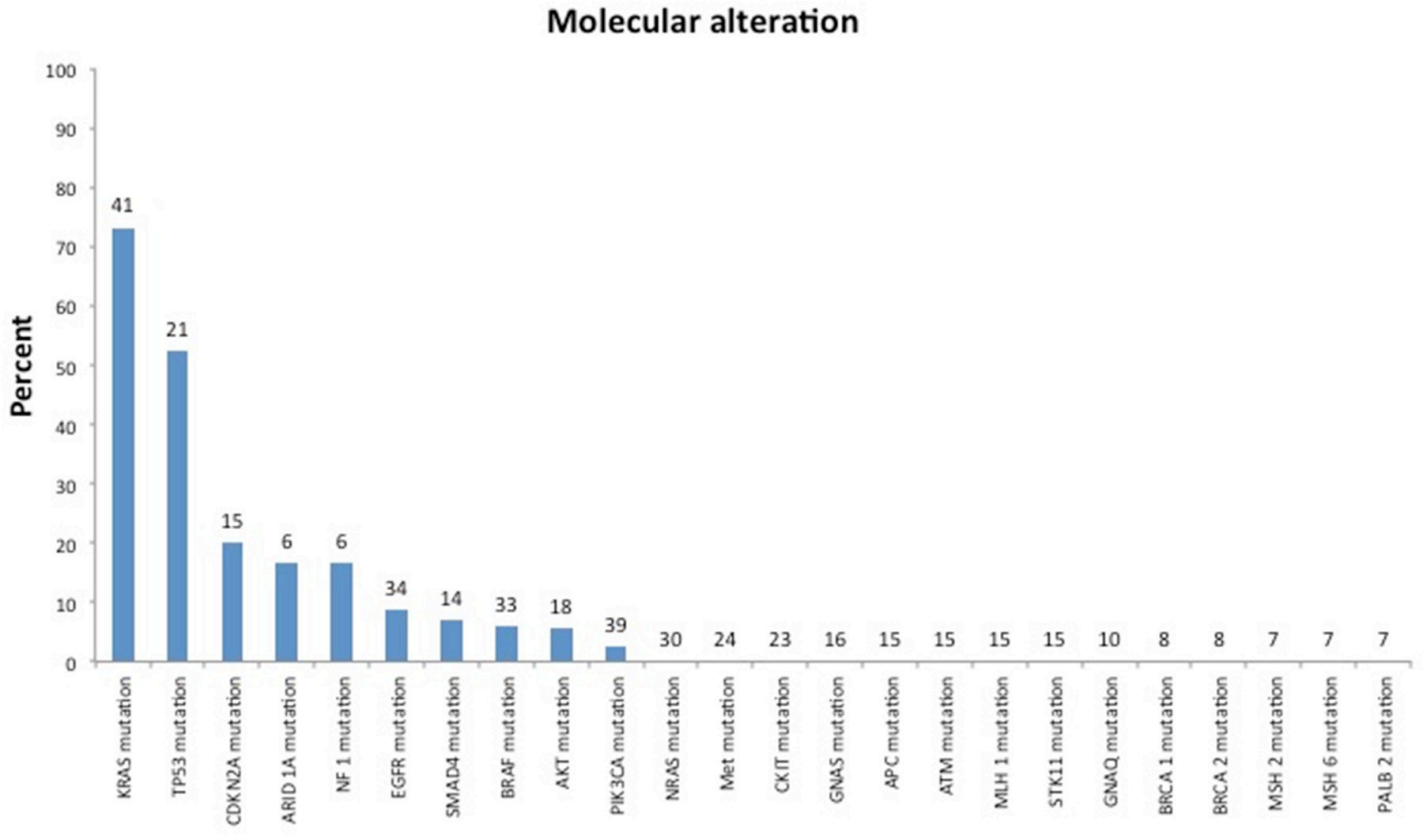
Mutation spectrum of patients tested for biomarkers with either panel or hotspot testing Number above bars indicate numbers of patients tested. Some genes tested by panels with no alteration found in the study cohort were not included.

Fifty-one patients (54%) were treated with targeted therapy alone or in combination with cytotoxic therapy. Of the 95 patients in the study, 56 (59%) had biomarker profiling performed. Biomarker status was determined using platforms from Foundation Medicine Illumina HiSeq 2000, Caris Molecular Intelligence Illumina MiSeq and Illumina TruSeq Amplicon cancer hot-spot panel, or the 46-gene or 50-gene Ion Ampliseq cancer panel (Life Technologies) (Figure 3B), and gene-specific hotspot testing were used to determine biomarker status (Figure 4). Thirty-one of 37 patients (84%) tested had *KRAS* mutations and 10 of 17 patients (59%) tested had *TP53* mutations. Three of 22 patients (14%) tested had *EGFR* mutations, 2 of 23 patients (9%) tested had *BRAF* mutations, 2 of 25 patients (8%) tested had *PIK3CA* mutations, 1 of 4 patients (25%) tested had *AKT* mutations, and 1 of 1 patients tested had an *FGFR3* mutation. Of these 56 patients, only 6 patients (10.7%) were placed on trials based on biomarker testing results (Figure 3). Of these 6 patients, 5 had progressive disease, and 1 had stable disease for more than 4 months, while enrolled in the biomarker-matched trial. The median OS from the date of initiation of the best phase I trial was 3.4 months (95% CI, 1.5-not reached) in the 6 patients whose treatment was based on biomarker testing results and 4.5 months (95% CI, 3.1-5.32.7 months) in the 89 whose treatment was not based on biomarker testing results.

## DISCUSSION

In this study, we examined whether outcomes of patients with pancreatic cancer treated on phase I trials have improved since the time of our previous analysis, which covered patients treated during 2004-2009 [11]. Since that time, there have been multiple breakthroughs in novel cytotoxics, targeted therapy, and immune therapy for cancer. We found a small, yet hopeful improvement in median OS from first consultation in the phase I clinic (from 5.0 months in our earlier study to 5.8 months in the current study). However, median OS from date of diagnosis was not improved (22.1 months in our earlier study and 20.6 months in the current study). Comparison of median OS from diagnosis across trials, however, must be interpreted with caution, because of guarantee-time bias, where analysis is timed from diagnosis to interpret a survival event during follow-up. This may be due to a time lag between the initation of treatment in one group of clinical trial patients or another [12].

Our findings from the current study indicate that chemotherapy, particularly in combination with targeted agents, continues to have a role in the treatment of pancreatic cancer. The median OS from the initiation of the first phase I trial was 5.2 months in patients treated with standard-of-care chemotherapeutic backbone agents compared to 4.2 months in patients treated with novel therapies. In our study, patients treated with anti-VEGF-based regimens had a slightly better OS, although this benefit was not statistically significant. Anti-VEGF therapy may act synergistically in combination with novel therapeutics to contribute to disease response, although thus far a benefit of anti-VEGF therapy has not been observed in larger phase III trials in pancreatic cancer.

Fifty-nine percent of patients in our current study had stage I-III disease at diagnosis, which may reflect a referral bias to our institution with the hopes of an operative approach to treatment in earlier-stage disease. Unfortunately, however, patients appeared to be referred to our phase I clinic relatively late in their disease course; only 3 patients were referred before metastasis was diagnosed, and the mean number of prior treatments at referral to our clinic was 3. Previous exposure to a large number of agents may increase the resistance of tumors to therapy and hinder the efficacy of novel drugs, although patients who had received no more than 3 prior regimens before treatment on the phase I trial had outcomes no better than those of patients who had received more than 3 regimens (Figure 2C). It is possible that with referral of treatment-naïve patients, we could see improved outcomes; however, data are currently lacking to support this approach.

FOLFIRINOX has replaced gemcitabine alone as the preferred agent for patients with metastatic pancreatic cancer and good performance status because of demonstration of the superiority of FOLFIRINOX in a study published in the *New England Journal of Medicine* in 2011. In 2014, the Food and Drug Administration approved nab-paclitaxel plus gemcitabine as a front-line option for advanced pancreatic cancer. As our patients were recruited from 2009 through 2014, there was a bias towards more patients receiving prior therapy with FOLFIRINOX over nab-paclitaxel plus gemcitabine. As more patients are treated with these regimens over time, one might expect a bias towards improved OS from diagnosis in our current study compared to our previous study published in 2011. However, patients in our current study with exposure to FOLFIRINOX or gemcitabine plus nab-paclitaxel prior to referral to our phase I clinic did not appear to have longer survival from date of diagnosis than patients not treated with these regimens (Figure 2D). In fact, in the multivariate analysis, patients who received prior FOLFIRINOX had significantly worse OS than those without prior FOLFIRINOX therapy. This may be because patients who have received prior FOLFIRINOX prior to referral to our phase I clinic have chemoresistant disease difficult to salvage after standard cytotoxic therapy. Additionally, patients who presented with a larger burden of disease up front may be treated with FOLFIRINOX by primary oncologists in the hopes of rapid relief of associated symptoms of disease.

Three patients in our study treated in phase I clinical trials had partial responses. Two of these patients were treated with regimens that included gemcitabine, nab-paclitaxel, and anti-VEGF therapy, and both survived more than 1 year from the date of diagnosis, one for 2.2 years after diagnosis. These 2 patients with partial responses received phase I therapy for 8.5 and 10.5 months, respectively. Although their survival may be attributed to treatment with the Food and Drug Administration-approved agents gemcitabine and nab-paclitaxel, their survival was at least equivalent to the reported mean OS of 8.5 months in the MPACT trial. The third patient with a partial response was treated with trametinib, and had a nearly 60% maximum reduction in tumor size by RECIST criteria; this patient received phase I therapy for approximately 11 months. Although our current study included a highly selected group of patients, there does appear to be a subset of patients with PDAC who will survive more than 1 year with indolent disease and good performance status. It should be noted, however, that these patients, with indolent disease, had been treated with 1-2 regimens prior to referral and none of these patients were treated with FOLFIRINOX or gemcitabine and nab-paclitaxel prior to phase I. Therefore these patients may be represented in the chemo-sensitive fraction and benefit from referral earlier on in treatment.

Data have shown that patients with pancreatic cancer and ascites tend to do poorly and have a shorter OS of less than 3 months [13]. In our study, the median OS of patients who presented with ascites was 3.8 months. We must be careful not to over treat patients in whom data suggest aggressive disease biology; such patients should instead be considered for referral to supportive care or hospice.

Nearly 60% of patients in our study underwent biomarker testing, but the proportion of patients who were matched to trials on the basis of biomarker results was rather low. Unfortunately, a majority of the alterations found in our cohort (including mutations in *KRAS* in 31 of 37 tested [84%] and *TP53* in 10 of 17 tested [59%]) are not currently actionable, and pancreatic cancer in general is known to have a heterogeneous array of alterations in infrequently mutated genes [14]. Furthermore, with the use of next-generation sequencing, it has been shown that pancreatic cancer has few actionable mutations when KRAS mutations are excluded [15]. Five actionable mutations were noted in our cohort (in *FGFR3*, *EGFR*, *BRAF*, *AKT*, and *PIK3CA*). Our limited data suggest that matching to biomarker-based therapy may improve outcomes, but no definitive conclusions can be made based on the outcomes of so few patients. This may make targeted therapy difficult in pancreatic cancer and speaks to a need for better understanding of underlying tumor biology along with therapeutic developments to match.

In our previous study, over half of the patients were treated in one specific trial. In the current study, patients were more evenly distributed between trials, allowing us to draw stronger conclusions about the influence of novel therapies in general. Our current study also included 4 trials with immune-based therapies, an exciting field in cancer medicine given the recent success of these agents in melanoma and lung cancer [16–18]. More recent studies have shown success of immunotherapy in microsatellite-high cancers, which may indicate some promise of such therapy in microsatellite-high pancreatic cancer [19]. Unfortunately, our results from the current study suggest that pancreatic cancer patients do not seem to benefit from immunotherapy. However our numbers are small, thus we cannot draw firm conclusions. An improved understanding of the immune system and development of novel therapeutic immune targets may result in better responses to immunotherapy in pancreatic cancer in the future.

This study was retrospective, which prevented us from gathering data on all endpoints and precluded firm conclusions. The retrospective design, however, does allow for a more temporal understanding of the disease process and effect of risk factors and patient characteristics on outcomes. Additionally, inherent to the phase I patient selection process is a bias towards selection of patients who are in better physical condition, as patients with poor performance status tend to be excluded from clinical trials. It is also difficult to draw conclusions regarding individual regimens given the small number of pancreatic cancer patients recruited per trial and unfortunately, none of the agents in our current study have made it to larger phase 3 clinical trials in pancreatic cancer; however, this remains one of the largest studies to date assessing the current status of novel agents for pancreatic cancer in the age of targeted therapy and immunotherapy.

In conclusion, the survival impact of recently developed cancer therapeutics in phase I clinical trials at MD Anderson remains unclear at this time. This indicates the continued need for pancreatic cancer research and development of innovative treatment options. At first glance, the short median progression-free survival of 1.9 months, which suggests that progression was often noted at the conventional time for the first restaging studies, after 2 cycles of treatment, may deter clinicians from enrolling patients with pancreatic cancer in phase I trials. However, the median OS of 4.5 months from initiation of first phase I treatment and the 1-year survival rate of 9% for patients treated on phase I studies, in this cohort in which many patients had received multiple prior chemotherapy or chemoradiation regimens, may argue in favor of referral to phase I trials for patients with good tumor biology and a reasonable performance status. However, this conclusion must be considered tentative and weighed against the creation of unrealistic expectations of a response. Biomarker-based therapies for pancreatic cancer have yet to prove successful despite gains in standard-of-care treatment and chemotherapy; however, subsets of patients with pancreatic cancer are living longer and seek novel options for treatment. Clinical trials built upon chemotherapy backbones with the addition of these novel agents in the first-line setting may show more success. It is imperative that we continue to develop innovative therapies for patients with this difficult-to-treat disease.

## MATERIALS AND METHODS

### Patient data collection

We reviewed the medical records of patients with pancreatic cancer who were treated in the Phase I Clinical Trials Program of the Department of Investigational Cancer Therapeutics at MD Anderson Cancer Center from January 2009 through December 2014. All trials were included that enrolled patients with a diagnosis of locally advanced or metastatic pancreas cancer. Patient demographics, stage at diagnosis, prior treatment history, and clinical outcomes were assessed. Data were collected from transcribed notes in the electronic database. Patient records were reviewed from the time of presentation at MD Anderson. Relevant records provided by outside healthcare providers were also reviewed. The study was approved by the MD Anderson Institutional Review Board.

To be eligible for participation in phase I clinical trials, patients with pancreatic cancer had to be at least 18 years of age and had to have metastatic or unresectable disease, measurable disease according to the Response Evaluation Criteria in Solid Tumors (RECIST)[20] or evaluable disease, performance status of 0 to 2, and a life expectancy of at least 3 months. Women of childbearing potential were required to have a negative pregnancy test prior to enrollment on a clinical trial, and patients of childbearing potential were required to use contraception. There were additional unique eligibility criteria that differed from trial to trial. All patients provided written informed consent before enrollment on a trial. Data were anonymized to protect the identities of subjects involved in the research. Phase I treatment was selected after clinical, laboratory, and pathologic data were reviewed and the diagnosis of PDAC was confirmed. The matching of patients to designated investigational treatments varied over time according to protocol availability, individual trial recruitment status, and eligibility requirements.

After initiation of an investigational therapy, patients were evaluated at intervals designated in the protocols of individual clinical trials. At each visit, a history was taken and a physical examination was performed, along with necessary laboratory and imaging studies. Patients were assessed for adverse events (the grade of the event and the likelihood that it was attributable to the study medication), compliance with protocol treatment, and clinical response.

### Statistical analysis

The OS and duration-on-study distributions were estimated using the Kaplan-Meier method. Point estimates of medians and probabilities and their associated 95% CI estimates are reported when appropriate. Multivariable Cox proportional hazards regression models were used to evaluate the effect of individual factors while simultaneously adjusting for additional covariates. All statistical analyses were performed using S+ (Spotfire S+ 8.2 for Windows, TIBCO Software Inc.), and statistical significance was defined as *P <* 0.05.

## SUPPLEMENTARY TABLE




