# Supplementary material for: Outcomes of phase I clinical trials for patients with advanced pancreatic cancer: update of the MD Anderson Cancer Center experience
Source: Oncotarget. 2017 Aug 3;8(50):87163–73. doi: 10.18632/oncotarget.19897 (PMC5675623; doi:10.18632/oncotarget.19897)
Supplement: Supplementary file 1 [file oncotarget-08-87163-s001.pdf]

Outcomes of phase I clinical trials for patients with advanced pancreatic cancer: update of the MD Anderson Cancer Center experience

SUPPLEMENTARY MATERIALS

Supplementary Table 1: Summary of best phase I clinical trials not described in Table 1.

See Supplementary File 1
