# Supplementary material for: Outcomes of phase I clinical trials for patients with advanced pancreatic cancer: update of the MD Anderson Cancer Center experience
Source: Oncotarget. 2017 Aug 3;8(50):87163–73. doi: 10.18632/oncotarget.19897 (PMC5675623; doi:10.18632/oncotarget.19897)
Supplement: Supplementary file 2 [file oncotarget-08-87163-s002.doc]

Supplementary Table 1: Summary of best phase I clinical trials not described in Table 1.

| Treatment | Mechanism of Action | No. of Patients  (%)  (N = 44) | Mean Time on Study, months |
| --- | --- | --- | --- |
| Crizotinib  Dasatinib | ALK, cMet inhibition  BCR/ABL, Src, cKit inhibition | 3 (0.07) | 2.77 |
| Irinotecan by HAI  Bevacizumab with or without  Oxaliplatin or  Cetuximab | Topoisomerase I inhibition  VEGF inhibition  DNA crosslinking  EGFR inhibition | 3 (0.07) | 6.52 |
| Nab-Paclitaxel BY HAI | Microtubule stabilization | 3 (0.07) | 2.4 |
| PBI-05204 | PI3K/mTOR inhibition | 3 (0.07) | 3.99 |
| cMet inhibitor |  | 2 (0.02) | 1.42 |
| Nab-Paclitaxel by HAI  Bevacizumab  Gemcitabine | Microtubule stabilization  VEGF inhibition  Nucleoside analog | 2 (0.02) | 6.43 |
| MK-3475 (pembrolizumab) | Anti-PD1 antibody | 2 (0.02) | 3.4 |
| Pimasertib | MEK inhibition | 2 (0.02) | 1.63 |
| PI3K inhibitor  Paclitaxel | Microtubule stabilization | 1 (0.01) | * |
| Bendamustine  Bevacizumab | Alkylating agent  VEGF inhibition | 1 (0.01) | 1.83 |
| PI3K inhibitor  MEK inhibitor |  | 1 (0.01) | 3.17 |
| BKM-120  GSK-1120212 | PI3K inhibition  MEK inhibition | 1 (0.01) | 1.47 |
| Liposomal coenzyme Q10 |  | 1 (0.01) | 0.6 |
| CNTO-328 | Anti-IL-6 antibody | 1 (0.01) | 2.2 |
| Crizotinib  Pazopanib | ALK, cMet inhibition  cKit, FGFR, PDGFR, VEGFR inhibition | 1 (0.01) | 1.73 |
| Dasatinib  Bevacizumab  Paclitaxel | BCR/ABL, Src, cKit inhibition  VEGF inhibition  Microtubule stabilization | 1 (0.01) | 3.4 |
| Autologous activated dendritic cells |  | 1 (0.01) | 2.2 |
| Docetaxel  GSK-1120212 (trametinib) | Microtubule stabilization  MEK inhibition | 1 (0.01) | 21.1 |
| Erlotinib  Cetuximab | EGFR inhibition  EGFR inhibition | 1 (0.01) | 1.83 |
| Everolimus  IL-1R antagonist | mTOR inhibition | 1 (0.01) | 1.2 |
| Oxaliplatin by HAI  Bevacizumab | DNA crosslinking  VEGF inhibition | 1 (0.01) | 1.33 |
| 5-FU/leucovorin | Thymidylate synthase inhibition, nucleoside analog |  |  |
| Gemcitabine  Dasatinib | Nucleoside analog  BCR/ABL, Src, cKit inhibition | 1 (0.01) | 5.83 |
| GSK-1120212 (Trametinib) | MEK inhibition | 1 (0.01) | 11.03 |
| Stat 3 inhibitor |  | 1 (0.01) | 1.87 |
| Lapatinib  Sirolimus | HER2/neu and EGFR inhibition  mTOR inhibition | 1 (0.01) | 2.07 |
| LY-2606368 | CHK1 inhibition | 1 (0.01) | 0.43 |
| Metformin  Temsirolimus | mTOR inhibition  mTOR inhibition | 1 (0.01) | 0.87 |
| miRNA-34a mimetic |  | 1 (0.01) | 1.33 |
| Dual P70S6K, AKT inhibitor |  | 1 (0.01) | 0.77 |
| Paclitaxel  Carboplatin  Vemurafenib | Microtubule stabilization  DNA crosslinking  BRAF inhibition | 1 (0.01) | 0.3 |
| Revlimid  5-FU/leucovorin  Oxaliplatin | E3 ubiquitin ligase modulation  Thymidylate synthase inhibition, nucleoside analog  DNA crosslinking | 1 (0.01) | 6.87 |
| Sirolimus  Vorinostat | mTOR inhibition  Histone deacetylase inhibition | 1 (0.01) | 1.87 |

5-FU, 5-fluorouracil; HAI, hepatic arterial infusion; mTOR, mammalian target of rapamycin.

* Patient did not finish first infusion owing to allergic reaction
